# Evidence-based Health Care Continuing Education Seminars Improve Academic Staff Knowledge and Attitudes in Saudi Arabia

**DOI:** 10.12669/pjms.293.3299

**Published:** 2013

**Authors:** Saad M. Bindawas

**Affiliations:** Saad M. Bindawas, PT, PhD, FHEA, College of Applied Medical Sciences, King Saud University, Riyadh, Saudi Arabia

**Keywords:** Evidence-based health care, Health profession education, Academic staff

## Abstract

***Objective: ***To assess the influence of a monthly evidence-based health care (EBHC) seminar series on academic staff knowledge, attitudes, and barriers regarding EBHC practice.

***Methodology: ***All academic staff in the College of Applied Medical Sciences (CAMS), King Saud University, Saudi Arabia, were sent a validated web-based survey. The survey contained 35 items regarding 3 domains: knowledge (14 items), attitudes (11 items), and barrier factors (10 items). A 5-point Likert scale was used and descriptive statistics were generated for demographic data and participants’ responses to each item.

***Results: ***Among academic staff at CAMS, 79 of 198 (40%) completed the survey. Among the survey respondents, 58% had attended at least one EBHC seminar. Those who had attended at least one seminar had better knowledge of 8 items compared with those who did not attend any seminars (*P*<.05). Academic staff members who attended at least one seminar were more likely to have a positive attitude regarding EBHC. Insufficient time was the only factor that significantly differed between the 2 groups, serving as a barrier to EBHC practice.

***Conclusions: ***Our findings suggest that EBHC seminars may improve the abilities and skills of academic staff for using EBHC.

## INTRODUCTION

Evidence-based medicine (EBM), or evidence-based practice, is the application of best-available evidence gained from scientific methods to clinical decision making.^1^ This approach helps clinicians understand whether a treatment will do more good than harm. Mainly implemented in the United States, EBM principles have been increasingly incorporated into medical curricula to enable students to learn how to use the best available evidence. To date, there has been little effort to adopt EBM principles in the Middle East, Asia, and Europe, but EBM has been taught in several schools of health professions.^2^

Teaching students of the health professions to become lifelong learners and to familiarize them with the workings of the health care system was the first step in introducing EBM into the curricula; the term later evolved into evidence-based health care (EBHC). In 1992, EBHC was introduced to the medical students at Albany Medical College in Albany, New York, through a course titled Comprehensive Care Case Study.^3^ Following that model, health professions students should learn that EBHC is the most ethical way to practice because it integrates up-to-date patient-oriented research into clinical decision making to improve patient outcomes.^4^

The CME program curriculum for health care professionals includes acquiring EBHC knowledge and skills.^5^ Health care professionals commonly use these programs to build their knowledge further; however, there is limited evidence to suggest that CME influences physicians’ clinical behavior.^6^ Several investigators have examined the effects of teaching EBHC to health care professionals in developed and developing countries.^7^-^9^ Feise and colleagues assessed knowledge of relevant subjects among American chiropractic practitioners in a workshop and found that continuous education(CE) was effective in enhancing EBHC knowledge.^10^ Johnston and colleagues used the self-reported Knowledge and Attitude Questionnaire^11^ and found that knowledge and attitude were improved with CE.

Increased education may help to encourage EBHC’s implementation in health care practice.^12^ To develop efficient EBHC learning opportunities for future health care professionals, it is essential to undertake a needs assessment and to evaluate their level of knowledge and attitudes. This study was designed to assess the impact of a monthly EBHC seminar series on academic staff knowledge, attitudes, and barriers regarding EBHC practice. We hypothesized that academic staff who attended at least one seminar would have more favorable responses than academic staff who did not attend any seminar.

## METHODOLOGY

During the 2010–2011 academic year, we distributed a validated web-based questionnaire on EBHC to all 198 academic staff in the College of Applied Medical Sciences (CAMS), King Saud University, Riyadh, Saudi Arabia. The questionnaire was a modified version from previous studies.^13^^,^^14^ It included 35 items in 3 domains—knowledge claim (14 items), attitudes (11 items), and barrier factors (10 items)—assessed on a 5-point Likert scale. We also collected academic staff demographic data.

No formal written informed consent was obtained; consent was assumed to be provided when participants completed the questionnaire. The CAMS Research and Ethics Committee approved the study.

***Data collection and statistical analysis: ***Data were analyzed using chi-square and/or *t *tests as appropriate to describe respondents’ sociodemographic and professional characteristics. Independent two-sample Student’s *t *tests were used to compare the means of the scores between those who attended and those who did not attend any seminar. Because the data were derived from a Likert scale, it was reanalyzed using the Wilcoxon–Mann–Whitney test (a nonparametric test analogous to the t-test) to further assess the results.^15^All analyses were performed using SAS version 9.1.3 (SAS Institute, Cary, NC). The significance level was set at *P *<0.05.

## RESULTS

***Participant characteristics: ***Seventy-nine of 198 (40%) CAMS academic staff members completed the survey. Among the respondents, 46 (58%) attended at least one EBHC seminar. Demographic characteristics of responding academic staff are presented in Table-I. The largest groups of respondents were Saudi nationals (36.7%) and men (34.2%) with a PhD or other clinical doctorate degree (35.4%).

***Knowledge about EBHC: ***Results regarding EBHC knowledge are presented in Table-II. Participant level of knowledge was significantly different between the two groupsfor8 items. Academic staff who attended at least one seminar had better knowledge about those 8 items than those who did not attend any seminar. Generally, academic staff who did not attend any CAMS seminar lacked adequate knowledge about basic EBHC concepts.

***Attitudes toward EBHC:*** Table-III compares 11 attitude-related items. There were only 3 significant discrepancies between those who attended and those who did not attend any of the seminars. Those who attended at least one seminar were more likely to have a positive attitude toward EBHC than the other group. Both groups felt that teaching EBHC foundations to CAMS students is important. Generally, the attitude towards EBHC was positive, and the most participants in both groups were more likely to take part in EBHC training courses.

***Obstacles regarding EBHC: ***When asked to indicate their perceived barriers to implementing EBHC, respondents most frequently cited a lack of EBHC training (Table-IV). Academic staff members who did not attend any seminar were most likely to cite the insufficient time factor as their reason for not attending the seminars. The Wilcoxon-Mann-Whitney test was used to confirm all results.

## DISCUSSION

This study demonstrated that all academic staff who attended seminars had better knowledge and a more positive attitude toward EBHC compared with those who did not attend any seminar. Additionally, the difference of level of knowledge was significant for 8 of the 14 items; however, the difference between groups on attitude was significant for only 3 among the 11 items as perceived by the academic staff. The insufficient time factor was found the only significant barrier to EBHC implementation, and that lack of training differed between the groups among the 10 items, indicating lack of training as an EBHC implementation barrier.

This study is descriptive and exploratory; it highlights areas of strength and weakness regarding EBHC knowledge and attitudes among CAMS academic staff. We believe this study can serve as a basis for future studies. The following are the highlights of our study: First, it grouped different skills regarding EBHC knowledge, attitudes, and barriers among different CAMS academic staff departments. Second, it revealed that a monthly seminar improves knowledge and positive attitudes toward EBHC among academic staff. Third, it shed light on the obstacles to EBHC implementation. 

**Table-I:** Demographic characteristics of 79 academic staff responding to the survey

*Variables*	*Attended* *(N = 46)*	*Did Not Attend* *(N = 33)*
*Age*		
20–29	11 (13.9%)	8 (10.1%)
30–45	25 (32.6%)	19 (24%)
Over 45	10 (12.7%)	6 (6.7%)
*Sex*		
Female	19 (24%)	19 (24%)
Male	27 (34.2%)	14 (17.8%)
*Nationality* ^a^		
Saudi	29 (36.7%)	13 (16.4%)
Non-Saudi	17 (21.5%)	20 (25.3%)
*Academic* *Rank*		
Junior academic staff	33 (41.8%)	28 (35.4%)
Senior academic staff	13 (16.5%)	5 (6.3%)
*Highest Degree Earned*		
Bachelor’s degree	3 (3.8)	8 (10.1%)
Master’s or other graduate degrees	15 (19%)	7 (8.9%)
PhD or other clinical doctorate degrees	28 (35.4%)	18 (22.8%)
*Academic Department*		
Biomedical technology	3 (3.8%)	4 (5%)
Clinical laboratory science	5 (6.3%)	5 (6.3)
Community health	10 (12.6%)	3 (3.8%)
Dental health	6 (7.6%)	2 (2.5%)
Optometry	7 (8.8%)	6 (7.6%)
Radiation sciences	4 (5%)	6 (7.6%)
Rehabilitation sciences	11 (13.9%)	7 (8.8%)
*Registered with the Saudi Commission for Health Specialties* ^a^		
Yes	14 (17.7%)	3 (3.8%)
No	32 (40.5%)	30 (37%9)

^a^ *P*<.05

**Table-II:** Knowledge comparison between academic staff who attended an evidence-based health care seminar and those who did not attend^a,b^

	*All* *(N = 79)* *Mean (SD)*	*Attended* *(N = 46)* *Mean (SD)*	*Did Not Attend* *(N = 33)* *Mean (SD)*	*P Value* ^c^
Absolute risk	3.47 (1.27)	3.7 (1.15)	3.15 (1.37)	.068
Clinical effectiveness	3.82 (1.13)	4.07 (0.93)	3.48 (1.3)	.033
Clinical practice guidelines	3.75 (1.09)	3.91 (0.98)	3.52 (1.2)	.124
Coincidence bias	2.61 (1.31)	2.89 (1.3)	2.21 (1.24)	.022
Confidence interval	3 (1.24)	3.24 (1.16)	2.67 (1.29)	.047
Heterogeneity	3.33 (1.3)	3.57 (1.15)	3 (1.44)	.066
Inverse interval	2.86 (1.15)	3.09 (1.09)	2.55 (1.18)	.041
Meta-analysis	3.06 (1.27)	3.3 (1.24)	2.73 (1.26)	.047
Number needed to treat	3.62 (1.1)	3.8 (1.05)	3.36 (1.14)	.084
Odds ratio	3.19 (1.33)	3.3 (1.3)	3.03 (1.38)	.376
Publication bias	3.35 (1.26)	3.5 (1.3)	3.15 (1.2)	.223
Randomized controlled trial	3.71 (1.19)	4.07 (1.14)	3.21 (1.08)	.001
Relative risk	3.54 (0.98)	3.78 (0.89)	3.21 (1.02)	.012
Systematic review	3.75 (1.08)	4.17 (0.82)	3.15 (1.12)	<.001

^a^Scale: 5 = Understand and could explain to others; 1 = Never heard the term

^b^The *t *test was used.           ^c^*P*<.05

**Table-III:** Attitude comparison between academic staff who attended an evidence-based health care (EBHC) seminar and those who did not attend^a,b^

	*All* *(N = 79)* *Mean (SD)*	*Attended* *(N = 46)* *Mean (SD)*	*Did Not Attend* *(N = 33)* *Mean (SD)*	*P Value* ^c^
EBHC is not as important for my profession as it is for other health care professions	2.86 (1.26)	2.61 (1.22)	3.21 (1.24)	.036
Application of EBHC is necessary for any health care practice	3.94 (0.94)	4.02 (0.98)	3.82 (0.88)	.337
I am familiar with the medical search engines (e.g., MEDLINE, CINAHL, Pedro).	3.72 (1.18)	3.87 (1.15)	3.52 (1.2)	.192
EBHC takes into account patient preferences	3.66 (0.93)	3.7 (0.81)	3.61 (1.09)	.691
Practice guidelines are available for topics related to my profession	3.48 (0.96)	3.59 (0.91)	3.33 (1.02)	.259
EBHC does not ignore clinical experience	3.66 (0.95)	3.85 (0.87)	3.39 (1)	.040
Teaching EBHC foundations for CAMS^d^ students is important	3.99 (0.94)	4.09 (0.94)	3.85 (0.94)	.270
Attending CME/professional development EBHC events (e.g., courses, seminars, workshops) is very important for me	3.9 (1.15)	4.13 (1.13)	3.58 (1.12)	.034
Teaching CAMS students the art of bedside clinical experience is more important than teaching them EBHC	3.13 (0.99)	3.24 (0.99)	2.97 (0.98)	.236
More training is needed to be an EBHC teacher	3.94 (0.98)	3.98 (0.95)	3.88 (1.02)	.663
CAMS seminars have increased my awareness about EBHC	3.72 (1.15)	3.91 (1.09)	3.45 (1.2)	.087

^a^Scale: 5 = Strongly agree; 1 = Strongly disagree.

^b^The *t* test was used.           ^c^*P*< .05           ^d^College of Applied Medical Sciences.

**Table-IV:** Evidence-based health care (EBHC) implementation obstacle comparison between academic staff who attended an EBHC seminar and those who did not attend

	*All* *(N = 79)* *Mean (SD)*	*Attended* *(N = 46)* *Mean (SD)*	*Did Not Attend* *(N = 33)* *Mean (SD)*	*P Value* ^c^
Inability to apply research findings to individual patients with unique characteristics	3.59 (0.82)	3.52 (0.81)	3.7 (0.85)	.359
Insufficient time	3.82 (0.81)	3.65 (0.87)	4.06 (0.66)	.020
Lack of collective support among colleagues in my facility	3.58 (0.93)	3.59 (0.96)	3.58 (0.9)	.957
Lack of generalizability of the literature findings to my patient population	3.59 (0.9)	3.65 (0.82)	3.52 (1)	.521
Lack of information resources	3.37 (1.03)	3.43 (0.91)	3.27 (1.18)	.511
Lack of interest	3.13 (1.18)	3.24 (1.23)	2.97 (1.1)	.311
Lack of research skills	3.41 (1.14)	3.41 (1.27)	3.39 (0.93)	.938
Lack of training in EBHC	4.03 (0.93)	4.04 (0.94)	4 (0.94)	.839
Lack of understanding of statistical analysis	3.78 (0.89)	3.8 (0.93)	3.76 (0.83)	.815
Poor ability to critically appraise the literature	3.38 (1.09)	3.41 (1.13)	3.33 (1.05)	.748

^a^Scale: 5 = Strongly agree; 1 = Strongly disagree

^b^The *t* test was used.           ^c^*P*< .05

A statistically significant difference between the two groups was observed in the evaluation of understanding of 8 knowledge variables: clinical effectiveness, coincidence bias, confidence interval, inverse risk, meta-analysis, randomized controlled trials, relative risk, and systematic review. Statistically significant differences were also observed for the 2 groups regarding their opinions on EBHC’s importance for their profession, EBHC and clinical experience, and the importance of attending CME events.

Academic staff knowledge regarding heterogeneity and odds ratios was lowest among those who did not attend any seminars. This group also was more likely to believe that teaching CAMS students the art of bedside clinical experience was more important than teaching EBHC, and cited a lack of training skill as a barrier to implementation. To better ensure quality in the health professions, it is essential to develop national evaluation or assessment tools to measure knowledge and attitudes to ensure that academic staff members have acquired required skills. No significant differences were noted between the groups regarding the other 23 variables. We believe that attending CE programs such as the EBHC monthly seminar series may improve knowledge and attitudes among academic staff.

In Saudi Arabia, as in other countries, little is known about academic staff attitudes toward EBHC.^16^^,^^17^ EBHC has been examined in primary health care centers and general hospitals in Dammam, in eastern Saudi Arabia. Physician attitudes regarding EBM were examined, and the study showed that physicians’ confidence in EBM had increased.^14^ In the Southwestern region of Saudi Arabia, studies showed that physicians or academic staff had different perspectives on an acceptable level of knowledge and positive attitude regarding EBM or EBHC.^18^^,^^19^ The main barrier to implementing EBHC in Saudi Arabia is a lack of knowledge and basic skills.^20^ These findings differ from those of studies conducted in developed countries, where the primary barrier to implementing EBHC was lack of time.^16^

The overall acceptance of this seminar was strong; academic staff members were assured anonymity and provided honest feedback. Their comments primarily indicated an appreciation for the preparation and implementation of the monthly seminars, but they criticized the workload and seminar times. The academic staff felt comfortable throughout the seminar and provided constructive comments for improvement.

This study’s limitations may be related to the fact that it involved the use of a web-based survey to collect data. This method has not been commonly used in Saudi Arabia. However, researchers conducting several studies in Saudi Arabia generally invite respondents to complete web-based questionnaires.^21^ Another limitation was that we assessed only CAMS academic staff working in different disciplines. As a result, this study does not represent all health profession colleges or universities in Saudi Arabia. Additionally, no control group was used for comparison. Without an intervention, we may have overlooked changes that could occur. Finally, participants were not randomly selected; they participated voluntarily. Overall, this method was found to more efficiently protect data and prevent data loss.

## CONCLUSION

This study represents the first effort to evaluate if a monthly seminar program can improve knowledge and attitudes among academic staff in the CAMS at King Saud University. Study results demonstrate differences in knowledge and attitudes between academic staff who attended a seminar and those who did not attend a seminar in their evaluation of 35 variables. These findings indicate a lack of EBHC training and a need to improve knowledge of topics such as heterogeneity, odds ratios, and priority assessment. EBHC strategies must be developed to help academic staff improve their knowledge, attitude, and ability to share their new skills with colleagues through the EBHC seminar program.
